# Understanding Discomfort in Order to Appropriately Treat Fever

**DOI:** 10.3390/ijerph16224487

**Published:** 2019-11-14

**Authors:** Mattia Doria, Domenico Careddu, Flavia Ceschin, Maria Libranti, Monica Pierattelli, Valentina Perelli, Claudia Laterza, Annarita Chieti, Elena Chiappini

**Affiliations:** 1 Family paediatrician, Italian Federation of Paediatric (FIMP), 00100 Roma, Italy; domenicocareddu@fimp.pro (D.C.); ceschinf.ped@libero.it (F.C.); maria.libranti@tin.it (M.L.); mopi@dada.it (M.P.); 2 Istituti di Ricovero e Cura a Carattere Scientifico (IRCCS) Istituto Stella Maris, Calambrone, 56128 Pisa, Italy; vperelli@fsm.unipi.it; 3 Sanitanova S.r.l., 70100 Bari, Italy; claudia.laterza@sanitanova.it (C.L.); annarita.chieti@sanitanova.it (A.C.); 4 Ospedale Pediatrico Universitario Meyer, Dipartimento di Scienze della Salute—Università degli Studi di Firenze, Strutture Organizzative Dipartimentali (SOD) Malattie Infettive, 50139 Firenze, Italy; elena.chiappini@unifi.it

**Keywords:** discomfort, children, fever, paracetamol

## Abstract

Although national and international guidelines on the management of childhood and adolescent fever are available, some inadequate practices persist, both from parents and healthcare professionals. The main goal of bringing children’s temperature back to normal can lead to the choice of inappropriate drugs or non-necessary combination/alternation of antipyretic treatments. This behavior has been described in the last 35 years with the concept of fever-phobia, caused also by the dissemination of unscientific information and social media. It is therefore increasingly important that pediatricians continue to provide adequate information to parents in order to assess the onset of signs of a possible condition of the child’s discomfort rather than focusing only on temperature. In fact, there is no clear and unambiguous definition of discomfort in literature. Clarifying the extent of the feverish child’s discomfort and the tools that could be used to evaluate it would therefore help recommend that antipyretic treatment is appropriate only if fever is associated with discomfort.

## 1. Introduction

Although national [1] and international [2] guidelines on the management of fever in childhood and adolescence are available, some inappropriate practices [3] persist, both on the part of parents and caregivers, and on the part of healthcare professionals and pharmacists. The concentration on managing the fever with the overriding goal of bringing the child’s temperature back to normal can lead to the choice of inappropriate drugs for managing the symptoms, such as steroids [3], or unnecessarily combining or alternating antipyretic treatments [4]. Over the past 35 years the concept of fever-phobia has come to be used to describe anxiety in the face of fever. This phobia is partly due to the overabundant and persistent spread of non-scientifically based information and this has increased even more by the use of social media. It is therefore always important for pediatricians to continue to provide appropriate information to parents in order to evaluate the onset of the signs and symptoms of a possible severe underlying condition, and in order to investigate the child’s degree of discomfort rather than just focusing on their temperature [5]. The authors agree with the guidelines that suggest febrile children are only treated in cases of discomfort. However, since there is no clear and unambiguous definition of discomfort in the literature, the approach to febrile children has always been mainly focused on the lowering of body temperature. Clarification of the extent of the feverish child’s distress, and tools which could be used to evaluate this, would therefore help to put the recommendation that antipyretic treatment is only appropriate if the fever is associated with discomfort into practice [6].

## 2. The Extent of the Problem

Fever is the most common symptom in pediatrics representing the main cause of telephone triage and the reason for more than 30% of all pediatric consultations [7].

Fever is defined as an increase in central body temperature above the normal limits which, according to the Italian Guidelines and in accordance with the practical definition provided by the World Health Organization (WHO), is equivalent to a temperature of between 36.5 and 37.5 °C measured at the axillae [8].

Paracetamol and ibuprofen are currently the only drugs recommended for the treatment of fever in childhood [7].

Paracetamol therefore remains the trusted antipyretic of Italian caregivers and pediatricians; the results of a survey conducted in 2012 indicate paracetamol as the first choice for the management of fever by Italian pediatricians (98.3%), preferably given orally [5]. This data was confirmed by a more recent survey, in which 82.3% of respondents (family and hospital pediatricians and caregivers) considered oral paracetamol as the first line drug for the management of fever, giving the reason for the choice as better tolerability compared to ibuprofen with the same efficacy [9].

The 2016 update of the Italian Society of Pediatrics’ guidelines for the optimal management of fever in childhood recommends the administration of paracetamol at a dose of 15 mg/kg every six hours up to a maximum of 60 mg/kg per day. In newborns and infants up to three months of age, a dosage of approximately 10 mg/kg/dose is appropriate, up to a maximum of 40 mg/kg/day. If ibuprofen is used, the same guidelines recommend a dosage of 10 mg/kg/dose and up to three doses per day (therapeutic dosage 20–30 mg/kg/day).

In a comparative study of the two drugs [10] at the doses recommended by the guidelines, namely ibuprofen at a dose of 10 mg/kg/dose and paracetamol at a dose of 15 mg/kg/dose, equivalent efficacy and tolerability was observed.

Despite the few specific data in the literature regarding the treatment of discomfort in children with fever [11,12,13,14], paracetamol appears to be the most indicated and recommended pharmacological tool, both in terms of safety and efficacy and in that it is able to reduce the child’s discomfort thus leading to early symptomatic improvement during febrile illness.

Although the idea that the increase in body temperature represents a physiological and beneficial mechanism for fighting infections is well accepted, infections being the most frequent cause of fever in childhood, the public still have an impaired perception of the "risks" related to fever. This perception involves not only parents/caregivers, but also doctors, pharmacists, and healthcare professionals.

The concern about an increase in temperature in the child may be understandable given the fact that it could, even if only potentially, be the sign of a serious condition. Excessive anxiety in the presence of a temperature rise is, however, unjustified and counterproductive and risks causing further stress in children subjected to intrusive measurements of temperature. It also leads to unnecessary, inappropriate, and sometimes risky therapeutic interventions in terms of health (increase in side effects).

Numerous studies show that the phenomenon of "fever-phobia" is the actual anxiety-inducing driver responsible for erroneous behavior [6] and the main obstacle to the application of the guidelines’ recommendations [8]. The phobia of fever is, moreover, a common problem internationally, independent of how the healthcare system is organized and the type of assistance provided. It is also a subject which all pediatricians, in every part of the world, are increasingly facing, despite the evidence now available on the subject. Although knowledge about the management of children with fever has improved over the years, behavior still conforms little with the guidelines: many parents/caregivers still use traditional physical means and administer antipyretic drugs with inappropriate indications and dosages [7]. On the other hand, the lack of any universally shared indications amongst scientific communities favors the spread, including among healthcare professionals, of practices which are not in line with the guidelines’ recommendations [7]. 

For example, although the Italian Guidelines do not recommend its use, there is no unanimous agreement in the literature on the use of physical means to lower fever [15]. Sponging with water or alcohol, immersion in cold water, cold enemas or the application of ice packs only reduce the temperature temporarily by a modest amount (−0.2 °C) as they do not act on the hypothalamic set point. In comparison, they risk producing equally serious side effects (irritability, crying, paradoxical increase in body temperature, shivers and chills, hypoglycemia, coma or death due to the use of ethyl or isopropyl alcohol) [16]. Nevertheless, these methods are still very widely practiced, often with the aim of containing the parent or caregiver’s anxiety.

This creates the universal need for a strong, unambiguous, and coherent message regarding the management of children with fever [17,18,19]. Specific educational programs are needed which focus on the importance of considering fever as a phylogenetic adaptive mechanism, and which help to define and identify the signs and symptoms of objective danger and so distinguish them from those which are less significant. The focus would thus be shifted from the extent of the fever to the child’s actual discomfort. Correct information must reach all those who deal with children; that is, doctors, healthcare workers, pharmacists, teachers, parents, and caregivers in general, changing the objective from the anxiety-generated one of achieving a normal temperature to that of the appropriate and rational management of the child’s general condition and comfort.

Since it is known that the behavior and beliefs of parents can be greatly influenced by the attitude of pediatricians, early educational interventions aimed at the correct management of febrile children in the primary care setting become fundamental [20]. 

## 3. Materials and Methods 

To address the issue in question, the principal target of primary healthcare, the narrative literature review methodology was used. A search was therefore conducted within the main medicine and related sciences database, Pubmed, in order to perform the following work, using the following search string:

*fever[MeSH Terms] AND children[MeSH Terms] AND discomfort[Title/Abstract] AND ("2000/01/01"[PDat]: "2019/06/24"[PDat])*.

A manual search was also carried out in parallel.

Specific filters were applied to the research: publications from the last 19 years, from 2000 to 2019, in English or Italian, which included only subjects aged between 3 months and 14 years. We decided to exclude the 0 to 3-month population because in this age group it is advisable to monitor the patient closely in cases of fever and undertake any evaluation in the hospital setting.

**Figure 1:**
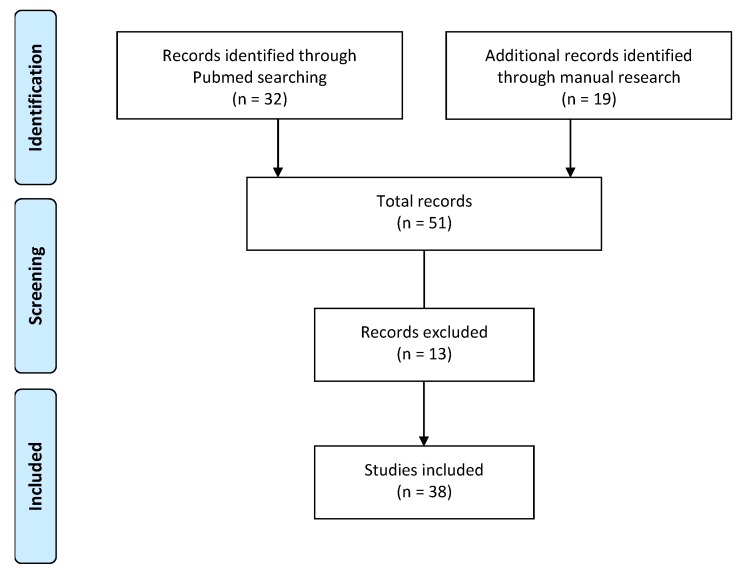
Flow chart showing identification, screening, and inclusion of studies for systematic review of discomfort in children.

Articles which concerned comorbidities such as cancer, cardiovascular disease, immunodeficiency, surgery and postoperative treatment, kidney or liver disease, typhoid fever, and tonsillectomy were also excluded (Figure 1).

## 4. Results

The literature search consists of both a Pubmed research and a manual search. The first is based on the inclusion of works with the definition of discomfort found through the string shown in Section 3 and excluding the population between 0 and 3 months and the presence of comorbidities such as cancer, cardiovascular diseases, immunodeficiency, surgery and treatment postoperative, renal or hepatic disease, typhoid fever, and tonsillectomy, and a manual search to identify further studies with the definition of discomfort not included by the string or other reasons for inclusion, as reported in the relevant column.

The results of our research led to 38 articles consistent with the inclusion and exclusion criteria, whose characteristics are detailed in the supplementary material in Table S1 (Pubmed research – Included articles), Table S2 (Pubmed research – Excluded articles) and Table S3 (Items included by manual search (excluding duplicates with Pubmed search)).

## 5. Discussion

Guidelines on the management of fever (Canada, France, USA, UK, Italy, WHO) agree on the need and importance of assessing the level of discomfort; this should be considered the only true rationale for symptomatic drug therapy.

However, there is no clear description of this condition in the literature and clinical experience shows that the degree of discomfort in illnesses, and in particular in those with fever, can vary greatly in its expression and intensity, from modest levels of distress to a marked sense of discomfort. A recent study showed that behavioral changes in the child are independent of the extent of the increase in body temperature; even with a very high temperature, some children generally continue to play as usual or show only a slight tiredness, whilst others show more significant signs of distress with more modest temperature rises [21]. 

The studies in the literature are not sufficient for identification of an unambiguous definition of discomfort, since the parameters considered and the environments in which they were studied were very variable. Most of the scientific works consider “generic” aspects, such as nervousness, annoyance, pain, fear, boredom, and tiredness [22] or refer to basic functions such as sleep, nutrition, and activity level [23]. Other authors also identify items such as lack of initiative or liveliness, the presence of mood disorders, moaning up to inconsolable crying or a reduction in social interaction and general loss of interest [10,24,25,26,27,28,29,30].

Almost all the studies examined use information gathered from the parents whilst there are relatively few attempts to construct observational scales to assess discomfort objectively. Discomfort is instead included as a parameter in studies on the assessment of pain in children in complex clinical conditions (oncological or chronic illnesses, acute conditions which require hospitalization or invasive investigations) [22]. One work [21] studied behavioral changes in children with fever and tried to identify the clinical components and their relationship with the fever. 

In addition, the studies which focused on fever in children mainly dealt with discomfort in relation to the methods of intervention and treatment without having provided an unambiguous definition. 

A quantitative evaluation of discomfort is obviously more complex than the detection of body temperature; the estimation and definition of discomfort in children with fever is unlikely to be simple, both because of the lack of clear and defined references and because of the risk of excessively subjective and unreliable interpretation. To this may be added an information asymmetry in the doctor–patient relationship. 

Studies in the field of developmental psychology may offer some references for identifying and defining the level of discomfort expressed by a child in the event of a febrile condition. The most frequent non-specific distress signals in children involve:

- behavioral changes;
- mood changes;
- alterations in the sleep–wake rhythm, in feeding, in activity level, in interest, in play;
- onset of symptoms of irritability and agitation, moaning, crying;
- withdrawal or dysfunction in social interactions.

What is really relevant in evaluating situations of distress is, however, a substantial change in a child’s performance, both with respect to typical developmental stages and in relation to their individual and temperamental characteristics [31]. 

The ways in which a child manifests his/her distress are linked to various factors which must be considered in the subjective evaluation: age, gender, level of cognitive development, cultural background, fear, beliefs and presentation of the illness, emotional experiences, personality, family members, the environment in which they live, and the reaction of their relations to their discomfort. There are also individual differences due to different sensitivities and different temperaments; if there are pain symptoms associated with the fever, the differences in emotional experiences, mental processes and behaviors instigated by each individual must be considered. In younger children it is more difficult to identify the symptoms of discomfort because of their objective difficulty in communicating distress (either because they are preverbal or because they are less able to explain their feelings) and it is therefore necessary to interpret more macroscopic and general signals. These are mostly behavioral and include, for example, irritability, reduced activity or hyperactivity, reduced appetite or changes in sleep–wake rhythm. In school-age children, in whom some clinical aspects can be more easily identified, including diffuse musculoskeletal pain or the headache which often accompanies the rise in temperature, emotional experiences or fears related to the illness which may interfere with warning symptoms should not be underestimated (minimization or exaggeration of discomfort or pain, fear of medical treatment).

The family also has a significant influence on the expression and evaluation of the child’s distress. Sociocultural factors may be relevant in interpreting the child’s distress but family and clinical history, parental needs and fears, emotional aspects, the family’s coping mechanisms, the presentation of illness, and finally the quality of the doctor–patient relationship may also affect how the discomfort is interpreted. 

Starting from these considerations, it is important to define what is meant by the discomfort of febrile children in a descriptive and functional sense and to evaluate whether this is measurable with a concise, practical tool, using language which is sufficiently communicative, and which can be used by pediatricians and understood by parents and caregivers. In this context it is necessary to bear in mind that the search for signals of discomfort, aimed at managing the fever, must be differentiated from the search for specific symptoms which pertain instead to the process of recognizing the cause of the fever and determining the clinical orientation and the specific therapeutic intervention, in order to guarantee a suitable clinical course and an early identification of emergency situations. Headache and arthromyalgia, being expressions of pain, will follow a specific management path, regardless of any link with a febrile state. It is also important to develop an approach which avoids the risk of over treating the discomfort medically.

Macroscopically, it appears to be useful to first of all consider parameters such as changes in the sleep–wake rhythm and changes in appetite, motor activity, mood, daily habits, and other signals; each aspect may include several further evaluations, which may facilitate communication with caregivers.

To this end, a table is presented below which is useful for recognizing the signs of discomfort in clinical practice (Table 1).

Improving the ability of parents and caregivers to recognize and monitor signs of discomfort may allow better management of the fever, achieving the dual objective of safeguarding its beneficial nature by avoiding inappropriate or unnecessary treatments and intervening, therefore, only in the event of discomfort which cannot be controlled by family care systems.

## 6. Conclusions

Developmental fever management guidelines suggest that febrile children should only be treated if they are uncomfortable. However, since studies in the literature do not allow the identification of an unequivocal and unambiguous definition of discomfort, the prevalent approach to febrile children is still mainly oriented towards the goal of lowering the temperature with the administration of the antipyretic beyond a cut-off point; this behavior is caused by the persistence of a wrong perception of the "risks" related to fever not only between parents/caregivers, but also between doctors, pharmacists, and health professionals.

By examining clinical trials and studies in the literature, some relevant behavioral indicators typical of discomfort can be isolated and can be identified in terms of a significant change in the child’s normal habits using information gathered by caregivers, such as changes in the sleep–wake rhythm and changes in appetite, motor activity, mood, daily habits, and other signals. 

**Figure 2:**
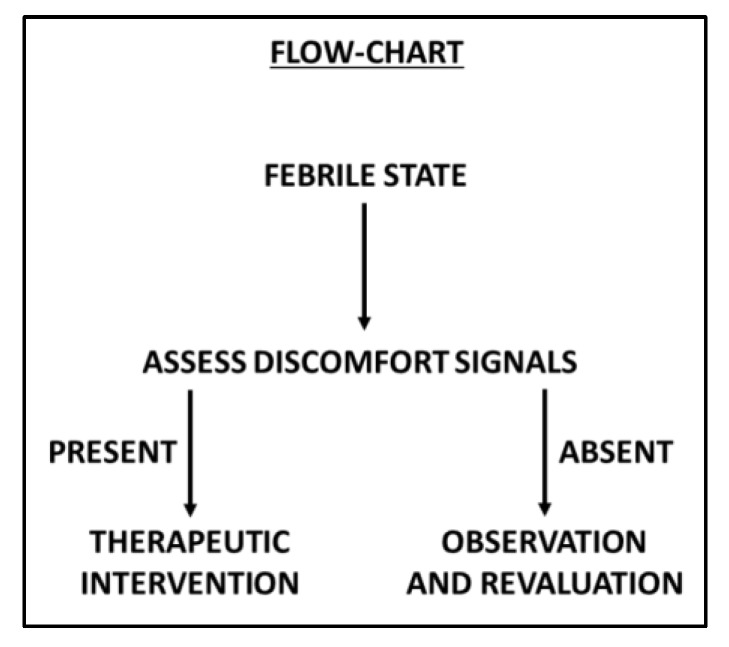
Flow chart showing the path of discomfort assessment.

The widespread involvement of all parties (doctors, pharmacists, health professionals, and caregivers) on the main objective of treating a child’s fever, namely, to alleviate fever-induced discomfort and not lower the body temperature beyond a certain limit, is essential [7]. Parents and health care professionals must be supported both in order to reduce the medical treatment of fever symptoms/signs in order not to risk treating the signs of discomfort when they can be managed and restrained by adequate family care and thus avoid use of drugs when not necessary (Figure 2).

## Figures and Tables

**Table 1:** Signals of discomfort.

**Signals of Discomfort**	**Variations of Sleep-Wake Rhythm**	delayed sleep phase
		early sleep phase
		night awakenings
	**Appetite Variations**	eat less
		no liquid intake
	**Variation in Motor Activity**	restlessness
		agitation
		weakness
		fatigue
	**Change in Mood**	irritability
		anger
		weeping
	**Variation in Daily Habits**	no play
		no interests shown
		seeking comfort
		uncooperative
	**Variation of Facial Expression**	changed look
		clenched teeth
		curled lips
		wrinkled forehead
		paleness/colour change
	**Other Signals**	tachypnoea
		chills
		widespread pain

## Supplementary Materials

The following are available online at https://www.mdpi.com/1660-4601/16/22/4487/s1, Table S1: Results of bibliographic research, Table S2: Pubmed research–Excluded articles, Table S3: Items included by manual search (excluding duplicates with Pubmed search).
