# Supplementary material for: Understanding Discomfort in Order to Appropriately Treat Fever
Source: Int J Environ Res Public Health. 2019 Nov 14;16(22):4487. doi: 10.3390/ijerph16224487 (PMC6888030; doi:10.3390/ijerph16224487)
Supplement: Supplementary file 1 [file ijerph-16-04487-s001.pdf]

International Journal of
Environmental Research
and Public Health

MDPI

Supplementary material

Table S1. Results of bibliographic research.

Pubmed research – Included articles								
**n°**	**Title**	**Authors**	**Journal/ Year of Publication**	**Type**	**Participants**	**Child's Age**	**Major Findings**	**Definition of Discomfort.**
1	Knowledge, attitudes and misconceptions of Italian healthcare professionals regarding fever management in children.	Chiappini E, Cangelosi AM, Becherucci P, Pierattelli M, Galli L, de Martino M.	BMC Pediatr. 2018	Research article	A questionnaire proposed to all the paediatricians attending the 2015 National Congress of Practice Paediatrics, held in Florence, Italy	Not available	Improvements in some pediatricians' misconceptions were observed over time. However, some incorrect habits persist.	Prolonged crying, irritability, reduced activity, reduced appetite, disturbed sleep
2	Effectiveness of paracetamol versus ibuprofen administration in febrile children: A systematic literature review.	Narayan K, Cooper S, Morphet J, Innes K.	J Paediatr Child Health. 2017	Systematic Review	Children	1 month to 12 years	There is little evidence supporting the superior efficacy of paracetamol or ibuprofen in the treatment of fever in children with indications that both drugs are equally effective.	Any pain, irritability, crying or stress observed in children
3	Sickness behavior in feverish children is independent of the severity of fever. An observational, multicenter study.	Corrard F, Copin C, Wollner A, Elbez A, Derkx V, Bechet S, Levy C, Boucherat M, Cohen R.	PLoS One. 2017	Observational, multicenter study	Children	6 months to 3 years	Sickness behavior and fever are two independent manifestations that are activated simultaneously during an infection.	Tendency to become irritated or angry, to complain, cry, or a change in facial expression
4	A clinical and safety review of paracetamol and ibuprofen in Children.	Kanabar DJ.	Inflammopharmacology. 2017	Review	Children	>3 months	Both paracetamol and ibuprofen have equally good safety and tolerability profiles, and when efficacy data is considered alongside safety, ibuprofen may be more preferable in providing relief from	Cough, cold, earache, fever

							discomfort, fever and pain.	
5	Italian Pediatric Society Panel for the Management of Fever in Children. 2016 Update of the Italian Pediatric Society Guidelines for Management of Fever in Children.	Chiappini E, Venturini E, Remaschi G, Principi N, Longhi R, Tovo PA, Becherucci P, Bonsignori F, Esposito S, Festini F, Galli L, Lucchesi B, Mugelli A, Marseglia GL, de Martino M	J Pediatr. 2017	Practice Guideline, Review	Children	Not available	Recent medical literature leads to reaffirmation of previous recommendations for use of antipyretics in children who are febrile.	Fever, local reactions, febrile convulsions
6	Healthcare professionals approach paediatric fever in significantly different ways and fever phobia is not just limited to parents.	Martins M, Abecasis F.	Acta Paediatr. 2016	Observational Study	A questionnaire administered to a sample of parents and nurses in the paediatric emergency rooms of two hospitals and to family doctors and paediatricians practising in Portugal.	Not available	The parents' and nurses' attitudes demonstrated fear of fever and its consequences. The approach to paediatric fever was significantly different among healthcare professionals.	Convulsions, irritability, dehydration
7	Cochrane in context: Combined and alternating paracetamol and ibuprofen therapy for febrile children.	Wong T, Stang AS, Ganshorn H, Hartling L, Maconochie IK, Thomsen AM, Johnson DW.	EvidBased Child Health. 2014	Cochrane Review	Six studies, enrolling 915 participants.	Not available	Both alternating and combined antipyretic therapies may be more effective at reducing temperatures than monotherapy alone. However, the evidence for improvements in measures of child discomfort remains inconclusive.	Pain, symptoms associated with fever, stress, days of nursery missed, reduced activity, reduced appetite and disturbed sleep.

8	A practical approach to the treatment of low-risk childhood fever.	Kanabar D.	Drugs R D. 2014	Review	Not available	Not available	Ibuprofen may provide greater efficacy in terms of the relief of symptoms in the distressed, feverish child and that short-term OTC ibuprofen and paracetamol have similar safety and tolerability profiles.	Reduced desire to eat or drink, symptom and general behaviour affected
9	Adherence among Italian paediatricians to the Italian guidelines for the management of fever in children: a cross sectional survey.	Chiappini E, D'Elios S, Mazzantini R, Becherucci P, Pierattelli M, Galli L, de Martino M.	BMC Pediatr. 2013	Cross sectional survey.	A questionnaire administered to paediatricians.	Not available	Behaviours of Italian paediatricians improved over time. However, some wrong attitudes need to be further discouraged, including use of physical methods and misuse of rectal administration.	Malaise, irritability, prolonged crying, signs of infection
10	Writing Committee of the Italian Pediatric Society Panel for the Management of Fever in Children. Management of fever in children: summary of the Italian Pediatric Society guidelines.	Chiappini E, Principi N, Longhi R, Tovo PA, Becherucci P, Bonsignori F,Esposito S, Festini F, Galli L, Lucchesi B, Mugelli A, de Martino M.	ClinTher. 2009	Research Support	Not available	Not available	Use of antipyretics—paracetamol (acetaminophen) or ibuprofen—is recommended only when fever is associated with discomfort.	Prolonged crying, irritability, reduced activity, reduced appetite and disturbed sleep
11	Paracetamol and ibuprofen for the treatment of fever in children: the PITCH randomised controlled trial.	Hay AD, Redmond NM, Costelloe C, Montgomery AA, Fletcher M, Hollinghurst S, Peters	Health TechnolAssess. 2009	Comparative Study, Multicenter Study, Randomized Controlled Trial	Children with fever > or = 37.8 degrees C and < or = 41 degrees C	6 months to 6 years	Young children who are unwell with fever should be treated with ibuprofen first, but the relative risks (inadvertently exceeding the maximum recommended dose) and benefits (extra 2.5 hours without fever) of using paracetamol plus ibuprofen over 24 hours should be	Pain, anguish, crying

		TJ.					considered.	
12	Comparative effectiveness of tepid sponging and antipyretic drug versus only antipyretic drug in the management of fever among children: a randomized controlled trial.	Thomas S, Vijaykumar C, Naik R, Moses PD, Antonisamy B.	Indian Pediatr. 2009	Comparative Study, Randomized Controlled Trial	150 children with axillary temperature 101F.	6 months to 12 years	Apart from the initial rapid temperature reduction, addition of tepid sponging to antipyretic administration does not offer any advantage in ultimate reduction of temperature; moreover, it may result in additional discomfort.	Crying, restlessness and irritability
13	Paracetamol plus ibuprofen for the treatment of fever in children (PITCH): economic evaluation of a randomised controlled trial.	Hollinghurst S, Redmond N, Costelloe C, Montgomery A, Fletcher M, Peters TJ, Hay AD.	BMJ. 2008	Multicenter Study, Randomized Controlled Trial	Children with fever > or = 37.8 degrees C and < or = 41 degrees C	6 months to 6 years	There is no strong evidence of a difference in cost between the treatments, but clinical and cost data together indicate that using both drugs together may be most cost effective over the course of the illness.	Reduced activity, reduced appetite and disturbed sleep
14	Paracetamol plus ibuprofen for the treatment of fever in children (PITCH): randomised controlled trial.	Hay AD, Costelloe C, Redmond NM, Montgomery AA, Fletcher M, Hollinghurst S, Peters TJ.	BMJ. 2008	Comparative Study, Multicenter Study, Randomized Controlled Trial	Children with fever > or = 37.8 degrees C and < or = 41 degrees C	6 months to 6 years	Young children who are unwell with fever should be treated with ibuprofen first, but the relative risks (inadvertently exceeding the maximum recommended dose) and benefits (extra 2.5 hours without fever) of using paracetamol plus ibuprofen over 24 hours should be considered.	Reduced activity, reduced appetite and disturbed sleep
15	Tepid sponging plus dipyrone versus dipyrone alone for reducing body temperature in febrile children.	Alves JG, Almeida ND, Almeida CD.	Sao Paulo Med J. 2008	Randomized Controlled Trial	Children with axillary temperature greater than 38 degrees C	6 months to 5 years	Tepid sponging plus dipyrone cooled faster during the first 15 minutes, but dipyrone alone presented better fever control over the two-hour period. Tepid sponging caused mild discomfort, crying and irritability for most of the children.	Crying, tremors, irritability
16	When the child has a fever.	BMJ Group.	Drug Ther Bull. 2008	Review	Not available	Not available		Myalgia or headache
17	'Fever phobia' in	Betz	Eur J	25-item	Caregivers	Not	Many caregivers	Malaise,

	the emergency department: a survey of children's caregivers.	MG, Grunfeld AF.	EmergMed. 2006	questionnaire		available	appear to confuse effects of fever with the harmful effects of hyperthermia. Aggressive and potentially dangerous home therapy and monitoring of fever is common among the caregivers surveyed.	vomiting
18	Childhood illnesses and the use of paracetamol (acetaminophen): a qualitative study of parents' management of common childhood illnesses.	Lagerløv P, Helseth S, Holager T.	FamPract. 2003	Research Support	Parents of pre-school aged children	Not available	Paracetamol constituted an important tool for parents in managing different upsets during childhood illnesses.	Pain
19	Evidence on the use of paracetamol in febrile children.	Russell FM, Shann F, Curtis N, Mulholland K.	Bull World Health Organ. 2003	Review	Not available	Not available	No evidence shows that it is beneficial to treat febrile children with paracetamol. Treatment should be given only to children who are in obvious discomfort and those with conditions known to be painful.	Painful conditions

Table S2. Pubmed research – Excluded articles.

n°	Title	Authors	Journal/ Year of Publication	Reason for Exclusion
1	Diagnostic accuracy of pediatric atypical appendicitis: Three case reports.	Wang ZH, Ye J, Wang YS, Liu Y.	Medicine (Baltimore). 2019	Appendicitis.
2	Principles, practices and knowledge of clinicians when assessing febrile children: a qualitative study in Kenya.	Hooft AM, Ripp K, Ndenga B, Mutuku F, Vu D, Baltzell K, Masese LN, Vulule J, Mukoko D, LaBeaud AD.	Malar J. 2017	Malaria.
3	Accuracy of tympanic and forehead thermometers in private paediatric practice.	Teller J, Ragazzi M, Simonetti GD, Lava SA.	Acta Paediatr. 2014	Discomfort due to minor distress associated with the use of digital ear thermometers.
4	Clinical and laboratory features of viral hepatitis A in children.	Blechová Z, Trojánek M, Kynčl J, Cástková J, John J, Malý M, Herrmannová K, Marešová V.	Wien KlinWochenschr. 2013	Hepatitis.

5	Pediatric urinary tract infection: does the evidence support aggressively pursuing the diagnosis?	Newman DH, Shreves AE, Runde DP.	Ann EmergMed. 2013	Urinary tract infections.
6	Are temporal artery temperatures accurate enough to replace rectal temperature measurement in pediatric ED patients?	Reynolds M, Bonham L, Gueck M, Hammond K, Lowery J, Redel C, Rodriguez C, Smith S, Stanton A, Sukosd S, Craft M.	J EmergNurs. 2014	Discomfort due to physical and psychological distress associated with the measurement of rectal temperature.
7	Symptomatic management of fever by Swiss board-certified pediatricians: results from a cross-sectional, Web-based survey.	Lava SA, Simonetti GD, Ramelli GP, Tschumi S, Bianchetti MG.	ClinTher. 2012	A questionnaire to describe the management of children with fever; lacks the definition of discomfort.
8	Performance of non-contact infrared thermometer for detecting febrile children in hospital and ambulatory settings.	Chiappini E, Sollai S, Longhi R, Morandini L, Laghi A, Osio CE, Persiani M,Lonati S, Picchi R, Bonsignori F, Mannelli F, Galli L, de Martino M.	J ClinNurs. 2011	Discomfort due to distress associated with the method of temperature measurement.
9	Clinical and radiological features of generalized lymphangiomatosis.	Wong CS, Chu TY.	Hong Kong Med J. 2008	Lymphangiomatosis.
10	Hyper-reactive malarial splenomegaly: rare cause of pyrexia of unknown origin.	Verma S, Aggarwal A.	Indian J Pediatr. 2007	Malarial splenomegaly
11	Symptomatic intravenous antipyretic therapy: efficacy of metamizol, diclofenac, and propacetamol.	Oborilová A, Mayer J, Pospísil Z, Korístek Z.	J Pain Symptom Manage. 2002	Cancer patients.
12	Management of the child with fever.	Robertson J.	Collegian. 2002	Comparison of the effectiveness of the single or combined use of sponging and paracetamol.
13	Antipyretics in children.	Chandra J, Bhatnagar SK.	Indian J Pediatr. 2002	Recommendations for the best way to control temperature.

Table S3. Items included by manual search (excluding duplicates with Pubmed search).

n°	Title	Authors	Journal/Year of Publication	Definition of Discomfort or Reason for Inclusion.
1	Fever Phobia: A Survey of Children's Parents in a Pediatric Outpatient Clinic.	Ae-Ran Choi and Jin Sun Kim	Child Health Nurs Res. 2014	Chills, hypoglycaemia, coma and death.
2	Evaluation of ibuprofen versus aspirin and paracetamol on efficacy and comfort in children	Autret E, Reboul-Marty J, Henry-Launois B,	Eur J Clin Pharmacol 1997	Crying, abnormal facial expression, abnormal behaviour of the child.

	with fever.	Laborde C, Courcier S, Goehrs JM, et al.		
3	Ibuprofen versus paracetamol in pediatric fever: objective and subjective findings from a randomized, blinded study.	Autret-Leca E, Gibb IA, Goulder MA	CurrMed Res Opin. 2007	Comfort understood as general behaviour and degree of relief. Equivalent efficacy and tolerability of ibuprofen (10 mg/kg) and paracetamol (15 mg/kg) in children with fever.
4	Symptomatic Management of Febrile Illnesses in Children: A Systematic Review and Meta-Analysis of Parents' Knowledge and Behaviors and Their Evolution Over Time.	N. Bertille, E. Purssell, N. Hjelm, N. Bilenko, E. Chiappini, E.G.P.M. de Bont, M.S. Kramer, P. Lepage, S.A.G. Lava, S. Mintegi, J.E. Sullivan, A. Walsh, J.F. Cohen, M. Chalumeau	Front. Pediatr. 2018	Fever-phobia.
5	Parental and medical knowledge and management of fever in Italian pre-school children.	Chiappini E, Parretti A, Becherucci P, Pierattelli M, Bonsignori F, Galli L, de Martino M.	BMC Pediatr. 2012	Dehydration and convulsions.
6	Guidelines for the symptomatic management of fever in children: systematic review of the literature and quality appraisal with AGREE II.	Chiappini E, Bortone B, Galli L, De Martino M.	BMJ Open. 2017	The main objective of treating a child's fever is to relieve the malaise induced by the fever and not the lowering of body temperature.
7	Paracetamol efficacy and safety in children: the first 40 years.	Cranswick N, Coghlan D.	Am J Ther. 2000	Paracetamol as first-line treatment for analgesia and antipyresis in children.
8	Twenty years of research on cytokine-induced sickness behavior.	Dantzer R, Kelley KW	Brain Behav Immun. 2007	Loss of appetite, drowsiness, withdrawal from normal social interactions, fever, joint pain and fatigue.
9	Recent Advances in Pediatric Use of Oral Paracetamol in Fever and Pain Management.	de Martino M, Chiarugi A.	Pain Ther. 2015	Paracetamol has been shown to have a lower risk of adverse events compared to NSAIDs and is indicated for use in children of all ages.
10	Role of paracetamol in treatment of childhood Fever: a double-blind randomized placebo-controlled trial.	Gupta H, Shah D, Gupta P, Sharma KK.	Indian Pediatr. 2007	Effective antipyretic effect of paracetamol with rapid improvement of symptoms in children with febrile illness without prolonging the duration of fever or excessive adverse effects.
11	Risks and benefits of paracetamol antipyresis in young children with fever of presumed viral origin.	Kramer MS, Naimark LE, Roberts-BraÈuer R, McDougall A, Leduc DG.	Lancet. 1991	Itching, reduced activity, reduced appetite.
12	Alternating antipyretics: antipyretic efficacy of acetaminophen versus acetaminophen alternated with ibuprofen in children.	Kramer LC, Richards PA, Thompson AM, Harper DP, Fairchok MP.	ClinPediatr (Phila). 2008	Significant reduction in fever with alternating paracetamol and ibuprofen regimen.
13	Physical methods for treating fever in children.	Meremikwu M, Oyo-Ita A.	Cochrane Database Syst	Temperature reduction by physical means.

			Rev. 2003	
14	Comparing efficacy and tolerability of ibuprofen and paracetamol in fever.	McIntyre J, Hull D.	ArchDis Child. 1996	Efficacy and tolerability of ibuprofen comparable to paracetamol in the treatment of fever in young children.
15	Fever and Pain Management in Childhood: Healthcare Providers' and Parents' Adherence to Current Recommendations.	Raffaeli G, Orenti A, Gambino M, Peves Rios W, Bosis S, Bianchini S, TagliabueC, Esposito S.	Int J Environ Res Public Health.2016	Sore throat associated with pharyngotonsillitis.
16	Antipyretic treatment in young children with fever.	Sarrell EM, Wielunsky E, Cohen HA	Arch Pediatr Adolesc Med. 2006	Effectiveness of alternating paracetamol and ibuprofen treatment regimen compared to monotherapy in fever reduction in children and newborns.
17	Beliefs and Expectations of Canadian Parents Who Bring Febrile Children for Medical Care.	Spiers JA, Enarson MC, Ali S, Vandermeer B, Wright RB, Klassen TP	Pediatrics 2012	Decreased interest in activities, energy, appetite, drinking and urination.
18	The child's perspective on discomfort during medical research procedures: a descriptive study.	Staphorst M., Benninga M., Bisschoff M., Bon I., Busschbach J., Diederen K., van Goudoever J., Haarman E., Hunfeld J., Jaddoe V., de Jong K., de Jongste J., Kindermann A., Konigs M., Oosterlaan J., Passchier J., Pijnenburg M., Reneman L., de Ridder L., Tamminga H., Tiemeier H. Timman R., van de Vathorst R.	BMJ Open, 2017	Nervousness, annoyance, pain, fear, boredom, tiredness.
19	Prescribing Controversies: An Updated Review and Meta-Analysis on Combined/Alternating Use of Ibuprofen and Paracetamol in Febrile Children.	Trippella G, Ciarcià M, de Martino M, Chiappini E.	Front Pediatr. 2019	The efficacy of combination or alternating therapy with paracetamol and ibuprofen in reducing body temperature compared to monotherapy does not appear to be clinically significant.
